# New inducible promoter for gene expression and synthetic biology in *Yarrowia lipolytica*

**DOI:** 10.1186/s12934-017-0755-0

**Published:** 2017-08-15

**Authors:** Marion Trassaert, Marie Vandermies, Fréderic Carly, Olivia Denies, Stéphane Thomas, Patrick Fickers, Jean-Marc Nicaud

**Affiliations:** 1 Micalis Institute, INRA, AgroParisTech, Université Paris-Saclay, 78350 Jouy-en-Josas, France; 2 Microbial Processes and Interactions, TERRA Teaching and Research Centre, University of Liège - Gembloux Agro-Bio Tech, Gembloux, Belgium; 3 Unité de Biotechnologies et Bioprocédés, Université Libre de Bruxelles, Brussels, Belgium; 4 Micalis Institute, INRA-AgroParisTech, UMR1319, Team BIMLip: Integrative Metabolism of Microbial Lipids, Bâtiment 526, domaine de Vilvert, 78352 Jouy-en-Josas, France

**Keywords:** Promoter, Regulation, Induction, Synthetic promoter, Erythritol, Erythrulose, Upstream activating sequence, *Yarrowia lipolytica*, Flow cytometry

## Abstract

**Background:**

The oleaginous yeast *Yarrowia lipolytica* is increasingly used as alternative cell factory for the production of recombinant proteins. At present, several promoters with different strengths have been developed based either on the constitutive pTEF promoter or on oleic acid inducible promoters such as pPOX2 and pLIP2. Although these promoters are highly efficient, there is still a lack of versatile inducible promoters for gene expression in *Y. lipolytica.*

**Results:**

We have isolated and characterized the promoter of the *EYK1* gene coding for an erythrulose kinase. pEYK1 induction was found to be impaired in media supplemented with glucose and glycerol, while the presence of erythritol and erythrulose strongly increased the promoter induction level. Promoter characterization and mutagenesis allowed the identification of the upstream activating sequence UAS1_EYK1_. New hybrid promoters containing tandem repeats of either UAS1_XPR2_ or UAS1_EYK1_ were developed showing higher expression levels than the native pEYK1 promoter. Furthermore, promoter strength was improved in a strain carrying a deletion in the *EYK1* gene, allowing thus the utilization of erythritol and erythrulose as free inducer.

**Conclusions:**

Novel tunable and regulated promoters with applications in the field of heterologous protein production, metabolic engineering, and synthetic biology have been developed, thus filling the gap of the absence of versatile inducible promoter in the yeast *Y. lipolytica*.

**Electronic supplementary material:**

The online version of this article (doi:10.1186/s12934-017-0755-0) contains supplementary material, which is available to authorized users.

## Background

Interest in non-conventional yeasts such as *Pichia pastoris, Hansenula polymorpha (Pichia angusta),* and *Yarrowia lipolytica* as cell factories for the production of recombinant proteins or biomolecules with biotechnological or pharmaceutical applications has increased over the years [1]. In *Y. lipolytica,* more than 100 heterologous proteins have been successfully produced at high yield, underscoring its production potential [1, 2]. *Y. lipolytica* is a model yeast species, well-known for its unusual metabolic properties such as the ability to grow on fatty acids or alkanes as sole carbon source and to accumulate intracellular lipids at high yield [3, 4]. This feature has enabled the development of metabolic engineering strategies to construct mutant strains to produce lipid for biodiesel and biojet fuel [5–12], or to synthetize unusual fatty acids [13], such as ω−3 [14], ricinoleic acid [15], conjugated fatty acids [16, 17], and fatty acid derivatives (e.g., fatty alcohol and dicarboxilic acid) [18]. Based on its ability to secrete large amounts of proteins and metabolites, *Y. lipolytica* has been used for several industrial applications, including heterologous protein synthesis, citric acid and erythritol production [19, 20]. Besides, *Y. lipolytica* has been accorded a GRAS (*generally recognized as safe*) status [19].

When developing an efficient cell factory, the choice of the promoter driving recombinant gene expression is crucial, and therefore represents one of the key parameters to be optimized. At present, few promoters have been identified and their regulation is not fully understood yet. Historically, the promoter from the *XPR2* gene, which encodes an alkaline extracellular protease, was the first to be characterized [21]. Although this promoter has been used successfully, its full induction requires high peptides concentrations and a pH above six, conditions that are often unfeasible at industrial scale. Comparison of strength and regulation of promoters from the glycerol-3-phosphate dehydrogenase (G3P), the isocitrate lyase (ICL1) and of genes involved in beta-oxidation pathway such as the 3-oxo-acyl-CoA thiolase (POT1) and the acyl-CoA oxidases (POX2, POX1 and POX5) was reported [22]. This provided the first strong promoters inducible by glycerol (G3P), ethanol (ICL) and oleic acid (POT1 and POX2). Other regulated promoters, such as the one from *LIP2* and *POX2* gene encoding an extracellular lipase and acyl-CoA oxidase 2, respectively, have been developed and characterized [23–25]. Using expression vectors based on *pLIP2*, higher protein productivities such as for Lip2p lipase have been obtained in *Y. lipolytica* than in other cell factories such as *P. pastoris*. Using the GAP constitutive promoter, Wang and colleagues [26] obtained lipase activity levels of 13,500 U/mL from a glucose fed-batch process in a 10-L bioreactor. In contrast, activity levels of 150,000 U/mL were obtained using *LIP2* promoter and a tryptone-olive oil fed-batch process [3]. However, the utilization of *pLIP2* and *pPOX2* is difficult in practice, especially in large-scale bioreactor, due to the hydrophobic nature (water insoluble) of the inducer (i.e. fatty acids or triglycerides). Other inducible promoters available in *Y. lipolytica* are those from genes encoding isocitrate lyase (pICL1, [22]), fructose-bisphosphate aldolase (pFBA1, [27]), phosphoglycerate mutase (pGPM) or glycerol-3-phosphate O-acyltransferase (pGPAT). They have been used for heterologous protein production with various successes (for a review see [1, 28]).

Constitutive promoters have also been considered. The functional dissection of *pXPR2* allowed the identification of one of its upstream activating sequence (UAS1_XPR2_) that is poorly affected by cultivation conditions [29]. Hybrid promoters, containing up to four direct repeats of UAS1_XPR2_ upstream of the minimal LEU2 promoter (*m*LEU2), were first constructed [30]. Among these, hp4d was widely used for heterologous protein production (for review see [2]). This latter has been at the basis of the *Y. lipolytica* YLEX expression kit commercialised by Yeastern Biotech Co. (Taiwan). More recently, an extended series of hybrid promoters, carrying various copy numbers (up to 32) of UAS1_XPR2_ upstream of *m*LEU2, were constructed [31]. Some of these hybrid promoters were shown to possess an efficiency eightfold higher than any known endogenous promoter from *Y. lipolytica* [31]. The promoter from the *TEF1* gene encoding the translation elongation factor-1α [32] is also widely used to drive constitutive gene expression in *Y. lipolytica*. Hybrid promoters with variable strengths derived from the latter were recently tested for the production of secreted proteins of industrial interest such as xylanase and glucoamylase [33]. This study highlighted that higher protein productivity does not necessarily rely on the strength of the promoter used for the expression of the corresponding gene.

In synthetic biology, gene expression must be fine-tuned in order to ensure optimal fluxes in the corresponding pathway or to avoid a metabolic burden. Hussain and colleagues [34] investigated promoter strength by shuffling promoter constitutive elements (UAS, proximal promoter, TATA box and core promoter) of various fungal gene promoters (TEF, POX2, LEU2, PAT1) in *Y. lipolytica.* They found out that engineering promoter architecture allows to modulate and to fine-tune gene expression level. However, to expend the range of this regulation, novel regulatory elements (UAS) and thus novel regulated promoters remain to be discovered.

In this study, we report on the identification of the inducible promoter from the *EYK1* gene encoding an erythrulose kinase in *Y. lipolytica*, the characterisation of its regulatory elements and the development of hybrid derivatives promoters showing different induction strengths and regulatory patterns depending on the genetic background of the recipient strain (WT or ∆*eyk1*). This set of novel promoters has direct applications for heterologous protein production, metabolic engineering and synthetic biology.

## Methods

### Growth and culture conditions

The *Y. lipolytica* strains used in this study were derived from the wild-type *Y. lipolytica* W29 strain (ATCC20460). The auxotrophic derivative Po1d (Leu^−^ Ura^−^) was previously described by Barth and Gaillardin [35]. *Escherichia coli* strain DH5α was used for hosting and amplification of recombinant plasmid DNA. All the strains used in this study are listed in Table 1. The media and growth conditions used for *E. coli* were described by Sambrook and colleagues [36]. YPD and YNB medium together with growth conditions for *Y. lipolytica* have been previously described by Barth and Gaillardin [35]. To meet auxotrophic requirements, uracil (0.1 g/L) and/or leucine (0.1 g/L) were added to the culture medium when necessary. Casamino acids (0.2% Bacto Casamino Acids, Difco, Paris, France), were added to bioreactors for faster growth rate. Growth of *Y. lipolytica* was performed in baffled 250 mL flask incubated at 28 °C at 160 rpm. YNB medium was supplemented with carbon source (10 g/L) as follows: glucose (YNBD), glycerol (YNBG), erythritol (YNBOL) or erythrulose (YNBOSE). Growth of ∆*eyk1* strains were performed in YNB medium with 0.25% glucose or glycerol as carbon source and 0.25% erythritol or erythrulose as inducer.

**Table 1:** List of strains and plasmids used in this study

Strain or (plasmid)	Genotype or other relevant characteristics	Source or reference
*E. coli*		
DH5α	ϕ*80dlacZ*∆*m15, recA1, endA1, gyrA96, thi*-*1, hsdR17 (r*_*k*_−, *m*_*k*_+*), supE44, relA1, deoR,* Δ*(lacZYA*-*argF)U169*	Promega
pCR4Blunt-TOPO^®^	Cloning vector, kanamycin	Invitrogen
pJET 1.2	Cloning vector, ampiciline	Thermo scientific
JME507	JMP113 (1.2 kb yl*LEU2* fragment, *LEU2*ex marker)	[41]
JME461	pRRQ2 (Cre ARS68 *LEU2*)	[53]
JME507	JMP113, *LEU2*ex	[41]
JME547	pUB4-CRE	[41]
JME803	JMP62-pPOX2-*URA3ex*	[54]
JME1427	JMP62-pTEF-*YFP*-*LEU2ex*	B Treton, unpublished, BIMLip/INRA
	JMP62-php4d-*YFP*-*URA3ex*	B Treton, unpublished BIMLip/INRA
JME2027	pCR4Blunt-TOPO—ClaI-4UAS1xpr2-BstBI	[33]
JME4123	PUC57-pEYK300A3B	GenScript, Hong-Kong
JME4124	PUC57-pEYK300A3b	GenScript, Hong-Kong
FCP007	pJET 1.2-pEYK300; *Cla*I-*Ba*mHI	This work
JME3934 (FCP013)	JMP62-pEYK300-*YFP*-*URA3ex*	This work
JME3994* (JMP3994)**	JMP62-pEYK450-*YFP*-*URA3ex*	This work
JME3988* (JMP3988)**	JMP62-pEYK300aB-*YFP*-*URA3ex*	This work
JME3991* (JMP3991)**	JMP62-pEYK300Ba-*YFP*-*URA3ex*	This work
JME3998* (JMP3998)**	JMP62-pHU4-EYK300-*YFP*-*URA3ex*	This work
JME4137* (JMP4137)**	JMP62-pEYK300A3B-*YFP*-*URA3ex*	This work
JME4139* (JMP4139)**	JMP62-pEYK300A3b-*YFP*-*URA3ex*	This work
*Y. lipolytica*		
W29	*MATA, wild*-*type*	[35]
Po1d	*MATA ura3*-*302 leu2*-*270 xpr2*-*322*	[35]
JMY330	Po1d, Ura^+^	[54]
JMY2101	Po1d, Leu^+^	[33]
JMY2900	Po1d, Ura^+^ Leu^+^	[33]
RIY147	Po1d *eyk1*::*LEU2ex*, Ura^−^	This work
RIY176	Po1d *eyk1*∆ Ura^−^ Leu^−^	This work
JMY2876	JMY330 + pTEF-*YFP*-*LEU2ex* (Ura^+^ Leu^+^)	B. Treton unpublished BIMLip/INRA
JMY6245 (FCY003)	JMY2101 + pEYK300-*YFP*-*URA3ex* (Ura^+^ Leu^+^)	This work
JMY6369	JMY2101 + pEYK300aB-*YFP*-*URA3ex* (Ura^+^ Leu^+^)	This work
JMY6372	JMY2101 + pEYK300Ab-*YFP*-*URA3ex* (Ura^+^ Leu^+^)	This work
JMY6375	JMY2101 + pEYK450-*YFP*-*URA3ex* (Ura^+^ Leu^+^)	This work
JMY6380	JMY2101 + pHU4-EYK300-*YFP*-*URA3ex* (Ura^+^ Leu^+^)	This work
JMY6681	JMY2101 + pEYK300A3B-*YFP*-*URA3ex* (Ura^+^ Leu^+^)	This work
JMY6684	JMY2101 + pEYK300A3b-*YFP*-*URA3ex* (Ura^+^ Leu^+^)	This work
RIY180 (JMY6637)	RIY176 + pEYK300-*YFP*-*LEU2ex* (Ura^−^ Leu^+^)	This work

* JME for the *E. coli* strain, ** JMP for the plasmid

### Growth in microplate and fluorescence analysis

*Yarrowia lipolytica* precultures were grown overnight in YNBD, before being centrifuged, washed with an equal volume of YNB medium without carbon source and resuspended in 1 mL of the same medium. 96-well microplates containing 200 μL of the appropriated medium (final volume) were inoculated with washed cells at an OD_600nm_ of 0.1. Growth was performed in a microtiter plate reader Synergy Mx (Biotek, Colmar, France) following the manufacturer’s instructions at 28 °C and 110 rpm. OD_600nm_ and fluorescence were measured every 20 min for 72 h. YFP fluorescence was analyzed with the wavelength settings ex: 505 nm/em: 530 nm. Fluorescence was expressed as specific fluorescence unit (SFU, normalized to biomass value) or mean specific fluorescence value (mSFU, mean value of SFU for the different sampling times). The SFU value of the wild-type strain JMY2900 (i.e. cell intrinsic fluorescence) was systematically deduced from that of the YFP reporter strain in the same experimental conditions (sampling time and medium). Cultures were performed in duplicates.

### Growth in bioreactor and monitoring of promoter induction by flow cytometry

*Yarrowia lipolytica* precultures were grown overnight in YPD, before being centrifuged, washed with an equal volume of YNB medium without carbon source and resuspended in 5 mL of the culture medium. The washed cells were used for bioreactor inoculation at an OD_600nm_ of 0.5. Chemostat were performed in 200 mL (150 mL working volume) DASGIP^®^ DASbox Mini Bioreactors SR0250ODLS (Eppendorf, Hamburg, Germany). A run of 7 h in batch mode was performed before being shifted in continuous mode with dilution rates as stipulated in the text. Feeding of fresh medium was ensured by a Watson Marlow 323S peristaltic pump (Watson Marlow, Falmouth Cornwall, UK), and removal of spent medium was ensured by a Watson Marlow 120U/DM3 peristaltic pump. Culture parameters were set as follows: temperature, 30 °C; agitation rate, 800 rpm; aeration rate at 1 vvm. Carbon source pulses (CSP) in the reactors were at fixed volume (4.2 mL), regardless of the pulse concentration. After each CSP, biomass, YFP fluorescence and carbon source concentrations were monitored for 8 h with a sampling frequency of 1 h. CSP were performed at steady state. Chemostat cultures were performed in duplicates.

YFP fluorescence was monitored using a BD Accuri™ C6 Flow Cytometer (BD Biosciences, NJ, USA). Flow rate was fixed at 14 µL/min, and samples were diluted with phosphate saline buffer (PBS) to reach a cell density ranging between 500 and 2500 cells/µL. For each sample, 40,000 cells were analyzed using the FL1-A channel to identify fluorescence associated with the YFP (excitation was performed with a 20-mW, 488-nm solid-state blue laser; the emission wavelength was 533/30 nm). Additionally, data from the forward scatter channel (FSC-A) were collected to get information on the size dispersion among the cell population. The flow cytometry dotplots (FL1-A/FSC-A) were analyzed using CFlowPlus software (Accuri, BD Bioscience). For further processing, the raw data were exported as.fcs files and loaded in MatLab using the fca_readfsc function (downloaded from the MatLab File Exchange file server; [37]). Background noise (cell intrinsic fluorescence) was fixed at 4000 fluorescence units. This value encompasses the fluorescence level of at least 99.3% of the wild-type cells (strain JMY2900) grown in YNBG (glycerol), YNBOL (erythritol) and of JMY6245 (pEYK300-YFP) grown in YNBG (glycerol). Relative fluorescence (RFU) was defined as the sample median fluorescence value minus the intrinsic fluorescence value. Proportion of induced cells refers to the number of cells showing a fluorescence signal higher than 4000 fluorescence units, relative to the total number of analyzed cells in the sample (i.e. 40,000). Gate Q1-UR of FSC-A/FL1-A cytograms encompasses induced cells.

### Plasmid and yeast strain construction

#### Plasmid construction

Restriction enzymes, DNA polymerases, and ligases were used in accordance with the manufacturer’s recommendations. Restriction enzymes were obtained from OZYME (Saint-Quentin-en-Yvelines, France). PCR amplifications were performed using an Eppendorf 2720 thermal cycler with PyroBest DNA polymerase (Takara) for cloning purpose and with GoTaq DNA polymerase (Promega) for deletion/overexpression verification. PCR fragments were purified using a QIAgen Purification Kit (Qiagen, Hilden, Germany), and DNA fragments were recovered from agarose gels using a QIAquick Gel Extraction Kit (Qiagen, Hilden, Germany). DNA sequencing was performed by GATC Biotech and primers were synthetized by Eurogentec (Seraing, Belgium). The Clone Manager software package (Sci-Ed Software) was used for gene sequence analysis and primer design. Disruption and expression cassettes were used to transform yeast cells using the lithium acetate method [38]. Transformants were selected on YNBcasa, YNBura, or YNB depending on their genotype. The genomic DNA from yeast transformants was obtained as described by Querol and colleagues [39]. Primers MT-URA3-for, MT-YFP-rev, pTEF-start, 61stop were used to verify successful insertion of the expression cassette and the promoter sequences. For each transformation, at least three independent transformants carrying the correct integration were analysed. The representative clones were used for this study.

The strains and plasmids used in this study are summarized in Table 1 and primers are listed in Table 2. The vectors carrying the yellow fluorescent protein (YFP) under the control of the pTEF and hp4d have been previously described (Table 1). The pEYK1 promoter and its derivatives (mutated and hybrid promoters) were introduced by exchange of the *Cla*I-*Bam*H1 region or the *Cla*I-*Spe*I region of YFP-encoding plasmid as described below.

**Table 2:** List of primers used in this study

Gene/names	Primers	Sequences	
P1-EYK	EYK-P-F	GTTGTGTGATGAGACCTTGGTGC	Deletion of EYK and verification of EYK deletion
P2-EYK	EYK-P-R-SfiI	AAAGGCCATTTAGGCCGCAGCTCCTCCGACAATCTTG	
T1-EYK	EYK-T-L-SfiI	TAAGGCCTTGATGGCCACAAGTAGAGGGAGGAGAAGC	
T2-EYK	EYK-T-R	GTTTAGGTGCCTGAAGACGGTG	
	LPR-L-SfiI	ATAGGCCTAAATGGCCTGCATCGATCTAGGGATAACAGG	
	LPR-R-SfiI	ATAGGCCATCAAGGCCGCTAGATAGAGTCGAGAATTACCCTG	
EYK-V1	EYK-V1	CGTACCCGAGATTGTACTGTTGTC	
EYK-V2	EYK-V2	CATAACCGCCTACCCTTGTAGC	
P300	pEYK300 F	GAC**ATCGAT**GCATCTACTTTTCTCTATACTGT	
P300	pEYK R	GAC**GGATCC**AGTAGATGTGTAAGTGTGTAGAAG	Promoter ampli, *Cla*I
P450	MT-TATAampli-F	ACG**ATCGAT**TTTGTGCAAGTGTGTGTGTGTG	Promoter ampli, *Bam*HI
P450	MT-TATAampli-R	ACG**ACTAGT**CAGGTCATCGGATTATGCAAGG	Promoter ampli, *Cla*I
ST043	pEYK-mut1	CGATGCATCTACTTTTCTCTATACTGTACGTTTCAATCTGGGGAAGCGGAATCCCAAAAG**GGAAAGCCG**CCGCATTAAGCTCCACAGCC	Promoter ampli, *Spe*I
ST044	pEYK-mut1BIS	CGATGCATCTACTTTTCTCTATACTGTACGTTTCAATCTGGGGAAGCGGAATCCCAAAAG**G** **ACGCGT** **CG**CCGCATTAAGCTCCACAGCC	WT bloc 1 (domain A)
ST045	pEYK-mut2	T**TGCATAATCCGA**TGACCTGA	Mutated bloc 1(domain a)
ST046	pEYK-mut2BIS	T**TG** **TACGCGTA** **GA**TGACCTGA	WT bloc 2 (domain B)
ST047	pEYK-mutA	GGCGTAATTCGAGGTGTCGGA**ACGTATTAGGCT**ACTGGACTGATC	Mutated bloc 2 (domain b)
ST048	pEYK-mutABIS	GGCGTAATTCGAGGTGTCGGA**AC** **ATGCGCAT** **CT**ACTGGACTGATC	WT blocA- complementary strand (domain B)
ST049	pEYK-mutB	TACGTAGATGAAAAGAGATATGACATGCAAAGTTAGACCCCTTCGCCTTAGGGTTTTC**CCTTTCGCG**	Mutated bloc A- complementary strand (domain b)
ST050	pEYK-mutBbis	TACGTAGATGAAAAGAGATATGACATGCAAAGTTAGACCCCTTCGCCTTAGGGTTTTC**C** **TGCGCA** **CG**	WT bloc B- complementary strand (domain A)
	MT-URA3-for	GCGTAGGTGAAGTCGTCAAT	Mutated bloc B- complementary strand (domain a)
	MT-YFP-rev	CAGATGAACTTCAGGGTCAGC	For promoter sequence verification (forward)
	pTEF-start	GGGTATAAAAGACCACCGTCC	For promoter sequence verification (reverse)
	61stop	GTAGATAGTTGAGGTAGAAGTTG	For gene verification (forward)
			For gene verification (reverse)

Modified sequence are in bold, restriction sites introduced are underlined

#### Construction of pEYK300

The promoter region of *EYK1* gene (pEYK300) was amplified from genomic DNA of *Y. lipolytica* strain W29 with primer pair pEYK300 F/pEYK R, designed to introduce *Cla*I and *Bam*HI restriction sites, respectively, in the amplified fragment. The resulting amplicon was purified and cloned into pJET1.2, to yield plasmid FCP007. The pEYK300 fragment was then released from FCP007 and cloned at the corresponding site of JMP1427, yielding the plasmid JMP3934.

#### Construction of pEYK450, pEYK300Ab and pEYK300aB promoters

Plasmid containing pEYK450 was obtained by PCR amplification of the intergenic region between genes YALI0F01628g and YALI0F01606g with primer pair MT-TATAampli-F/MT-TATAampli-R. This resulted in a 252 bp fragment carrying T, A and B boxes within a *Cla*I-*Spe*I fragment (sites added at the 5′ and 3′ ends, respectively). This fragment was ligated into FCP013 digested by *Cla*I-*Spe*I, to yield the plasmid JMP3994 (pEYK450).

Plasmids containing pEYK300Ab and pEYK300aB were obtained by exchange of the *Cla*I-*Spe*I fragment of JMP3934 (pEYK300) by two *Cla*I-*Spe*I DNA fragments carrying the A (aB) or B (Ab) mutated regions, respectively. They were obtained by annealing oligonucleotides ST044/ST045/ST050/ST047 (fragment aB) and ST043/ST046/ST049tB/ST048 (fragment Ab) (Table 2). The oligonucleotides ST044 and ST046 contain a *Mlu*I site for the verification of the insertion of the mutation. The resulting plasmids were designated JMP3988 (pEYK300aB) and JMP3991 (pEYK300Ab), respectively.

#### Construction of hybrid pHU4EYK300 promoter

The fragment carrying four tandem repeats of the UAS1_XPR2_ (HU4 deriving from hp4d) was obtained by *Cla*I-*Bst*BI digestion from the JMP2027 vector [33]. After gel purification, it was then ligated at the *Cla*I site of JMP3934 (previously digested by *Cla*I and dephosphorylated). Correct orientation of the HU4 region was verified by *Cla*I-*Bam*HI restriction and DNA sequencing. The resulting plasmid was named JMP3998 (pHU4EYK300).

#### Construction of hybrid EYK promoter

Synthetic promoters carrying three repeated of domains of A box upstream of the wild-type B box (A3B, JMP4123) and the mutated B box (A3b, JMP4124) were synthesised by GenScript Biotech Co. (China) with *Cla*I and *Spe*I sites at the 5′ and 3′ ends, respectively. The *Cla*I-*Spe*I fragments from JMP4123 and JMP4124 were ligated into JMP918 digested by *Cla*I-*Spe*I, yielding the plasmids JMP4137 (pEYK300A3B) and JMP4139 (pEYK300A3b), respectively.

#### Deletion of the EYK1 gene

The *EYK1* disruption cassette was generated by PCR amplification according to Vandermies and colleagues [40]. First, the upstream (Up) and downstream (Dn) regions of the *EYK1* gene were amplified using *Y. lipolytica* W29 genomic DNA as the template with the EYK-P-F/EYK-P-R-SfiI and EYK-T-L-SfiI/EYK-T-R as primer pairs. *URA3ex* marker was amplified from JME803 with the primer pair LPR-L-SfiI/LPR-R-SfiI (Table 2). Amplicons were digested with *Sfi*I before being purified and ligated, using T4 DNA ligase. The ligation product was amplified by PCR using the primer pair EYK-P-F/EYK-T-R. The *eyk1::URA3ex* disruption cassette was finally used to transform *Y. lipolytica* strain Po1d. The resulting strain was designated RIY147 (*eyk1::URA3ex,* Leu^−^). The auxotrophic derivative RIY176 was isolated after transformation of RIY147 with the replicative plasmid pRRQ2, according to Fickers and colleagues [41], for marker rescue (Table 2). The primers EYK-V1 and EYK-V2 (Table 2) were used for gene disruption verification.

### EYK1 promoter sequence analysis

Multiple alignments of nucleotide sequence of *EYK1* gene promoters among the Yarrowia clade: *Y. lipolytica* (YALI), *Yarrowia phangngensis* (YAPH), *Yarrowia yakushimensis* (YAYA), *Yarrowia alimentaria* (YAAL), and *Yarrowia galli* (YAGA) were performed using clustalW2 software [42] according to Larkin and colleagues [43]. Genome sequences of *Yarrowia* species were assembled and annotated by Cécile Neuvéglise, Hugo Devillers and coworkers (to be published). Homologues of YALI0F01606g in Yarrowia species were identified by Blast on the private site of GRYC (Genome Resources for Yeast Chromosomes; http://gryc.inra.fr) using YALI0F01606g gene as template. Promoter regions were retrieved using the download functionality developed by H. Devillers.

### Analytical methods

Cell growth was monitored by optical density (OD_600nm_). Erythritol, erythrulose, glucose and glycerol concentrations in the culture supernatant were measured by HPLC (Agilent Technologies 1200 series) using an Aminex HPX-87H ion exclusion column (Biorad 300 × 7.8 mm). Elution was performed using 15 mM trifluoroacetic acid as the mobile phase at a flow rate of 0.5 mL/min and a temperature of 65 °C. Erythritol, glucose and glycerol were detected using a refractive index detector (RID, Agilent Technologies), while erythrulose was measured at 210 nm with a UV detector (Agilent Technologies).

## Results and discussion

### EYK1 promoter is induced by erythritol and erythrulose

**Fig. 1:**
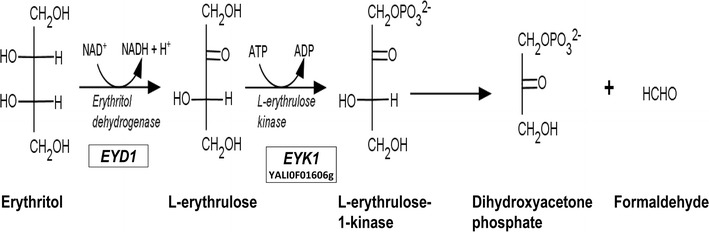
Erythritol catabolism pathway in *Yarrowia lipolytica*. Based on [45] and [46]. Erythritol is converted into l-erythrulose by an erythritol dehydrogenase encoded by *EYD1*, then l-erythrulose is phosphorylated by the l-erythrulose-1-kinase encoded by *EYK1* (YALI0F01606g) into l-erythrulose-1-phosphate

To date, two different pathways have been reported for erythritol catabolism. In a first one, erythritol is phosphorylated into erythritol-phosphate and then oxidized in erythrulose-phosphate [44]. In a second one, erythritol is first converted into erythrulose before being phosphorylated into erythrulose-phosphate [45]. We have recently identified and characterized *EYK1* gene (YALI0F1606g) [46] in *Y. lipolytica*. Disruption of the latter abolished yeast growth on erythritol medium, showing that *EYK1* gene is involved in erythritol catabolism. In addition, a ∆*eyk1* mutant was found to accumulate l-erythrulose. From this, it has been concluded that *EYK1* encode an erythrulose kinase (Eyk) and that erythritol catabolism in *Y. lipolytica* follows the pathway depicted in Fig. 1.

**Fig. 2:**
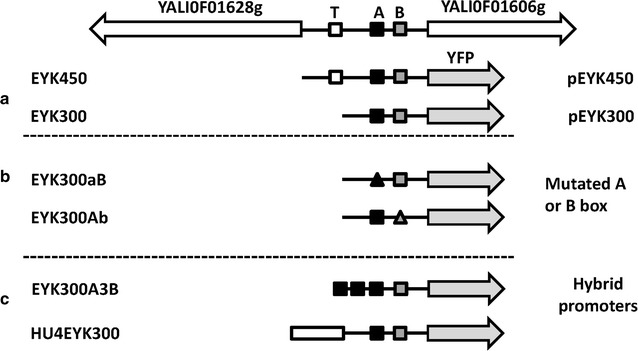
Schematic representation of promoters used in this study. Schematic representation of the genomic locus containing the upstream gene YALI0F01628g and the *EYK1* gene, YALI0F01606g. **a** Schematic representation of the native promoters pEYK_450_ (TATA box + native *A* box + native B box) and pEYK_300_ (native *A* box + native B box) controlling the expression of YFP; **b** Schematic representation of the mutated promoters pEYK_300_aB (mutated *A* box + native B box) and pEYK_300_Ab (native *A* box + mutated *B* box) controlling the expression of YFP; **c** Schematic representation of the hybrid promoters pEYK_300_A3B (3 *A* boxes + native B box) and pHu4EYK_300_ (4 tandem copies of UAS1_xpr2_ + native A box + native B box) controlling the expression of YFP.* Symbols* are *white filled square*: TATA box (*T*), *black filled square*: A box (*A*), *grey filled square*: B box (*B*), *black filled triangle*: mutated A box, *grey filled triangle*: mutated B box, *grey rightwards arrow*: YFP gene,* rectangle*: four tandem copies of UAS1xpr2

We therefore expect that *EYK1* gene expression is regulated by erythrulose, the substrate of Eyk, and/or by erythritol. To assess the regulation of the *EYK1* promoter, two fragments of 450 and 300 bp, (EYK450 and EYK300, respectively), corresponding to the intergenic region of genes YALI0F01606g and YALI0F01628g were used to construct a reporter gene system based on a yellow fluorescent reporter protein (YFP) (Fig. 2). Indeed, the YFP fluorescence was used to quantify the promoter induction level.

**Fig. 3:**
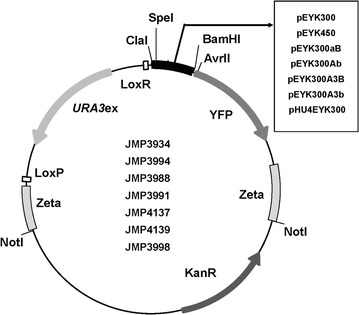
Schematic representation of plasmids constructed in this study. Plasmid pEYK300 contained the yellow fluorescent protein YFP, under the 285 bp promoter region of the *EYK1* gene (erythrulose kinase; YALI0F01606g). The vectors contain the zeta sequence for targeting the expression cassette obtained after *NotI* digestion. Kan^R^ and *URA3* markers are for selection in *E. coli* and *Y. lipolytica*, respectively. The *URA3* is flanked by *LoxP/LoxR* region for marker rescue (excisable marker *URA3*ex). JME3934 (pEYK300-YPF); JME3994 (pEYK450-YFP); JMP3988 (pEYK300aB-YPF); JMP3991 (pEYK300Ab-YPF); JMP4137 (pEYK300A3B-YPF)-YPF; JMP4139 (pEYK300A3b-YPF) and JMP3998 (pHU4EYK300-YPF)

Fragments EYK450 and EYK300 that span over 438 and 291 bp upstream of the *EYK1* start codon (Fig. 2a), were cloned in JMP1427 as described in Materials and methods to yield plasmids JMP3934 (pEYK300) and JMP3994 (pEYK450), respectively (Fig. 3). They were then used to transform *Y. lipolytica* strain JMY2101. Several independent transformants (3–6) were randomly selected for each construct and the corresponding YFP fluorescence measured during cell growth on erythritol medium (YNBOL). Since no differences in YFP fluorescence level, and thus promoter induction, could be observed (data not shown), one transformant of each construct was used for further studies, namely strains JMY6245 (pEYK300-YPF) and JMY6375 (pEYK450-YFP), respectively (Table 1).

**Fig. 4:**
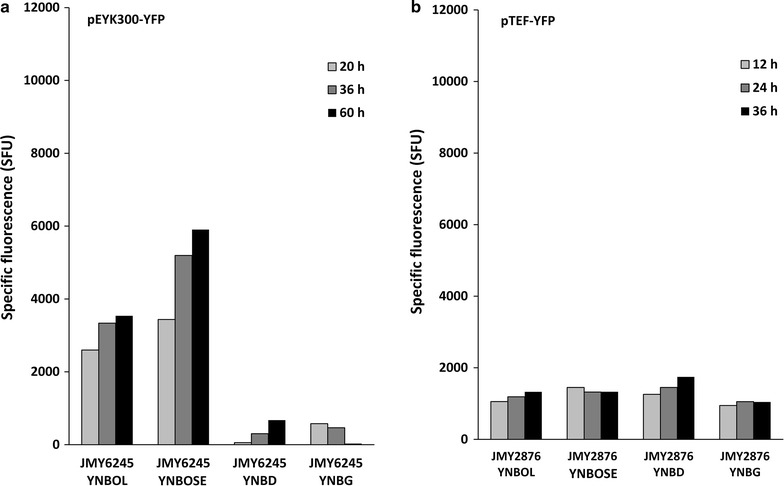
Time course of YFP fluorescence depending on culture medium for native *EYK1* and pTEF promoters. Specific fluorescence (SFU) corresponding to the expression of YFP under: **a** pEYK300 (JMY6245) and **b** pTEF (JMY2878). Growth in minimum media YNB containing 1% of specified carbon source (*OL* erythritol, *OSE* erythrulose, *D* dextrose, *G* glycerol)

Cell growth and YFP fluorescence were quantified over time during culture of strain JMY6245 in YNB minimal media supplemented with glucose (YNBD), glycerol (YNBG), erythritol (YNBOL) and erythrulose (YNBOSE). In medium containing erythritol (YNBOL) and erythrulose (YNBOSE), YFP fluorescence, and therefore pEYK300 induction levels, were significantly higher than in the presence of glucose (YNBD) and glycerol (YNBG) (3157 and 4844 mSFU as compared to 344 and 357 mSFU, respectively) (Fig. 4a). This clearly highlights that erythrulose and erythritol positively regulate pEYK300 induction by contrast to glucose and glycerol. However, the low fluorescence levels observed in YNBD and YNBG medium suggest that pEYK300 is slightly induced by glucose and glycerol. After 60 h of culture, the fluorescence level in medium supplemented with erythrulose was 1.5-fold higher than in the presence of erythritol (3536 and 5904 SFU, respectively). This suggests that erythrulose could be a better inducer than erythritol. Experiments performed with strain JMY6375 (pEYK450-YFP) in the same experimental conditions yielded similar results (data not shown). Therefore, the pEYK300 promoter seems to encompass the different regulatory elements requested for gene expression (UAS and URS). Consequently, pEYK450 promoter was not further analysed and we focused only on pEYK300 promoter in further experiments.

In order to assess the strength of pEYK300 induction by erythritol and erythrulose, it was compared to the strength of the strong constitutive pTEF promoter. YFP fluorescence of strain JMY2876 (pTEF-YFP) was measured in the same experimental conditions and compared to that of strain JMY6245. As shown in Fig. 4b, pTEF expression was similar in the four media tested, with fluorescence values being 1192, 1369, 1485 and 1016 mSFU in YNBOL, YNBOSE, YNBDD and YNBG, respectively. Expression levels for pEYK300 in YNBOL and YNBOSE were in average 2.6- and 3.5-fold higher than the expression level of pTEF, respectively.

### Identification of *EYK1* regulatory elements

**Fig. 5:**
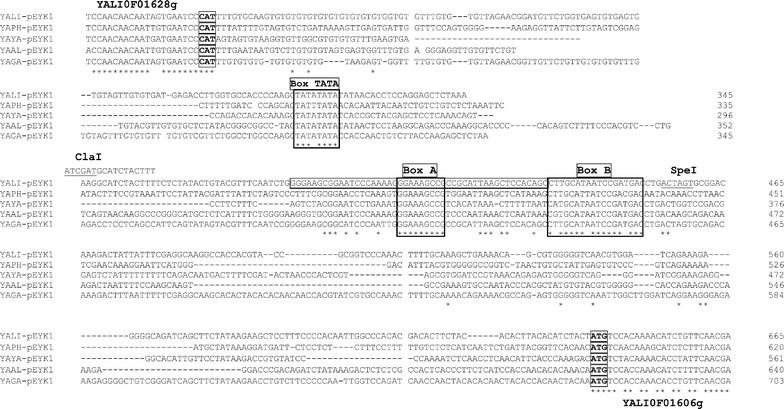
Multiple alignment of EYK promoter. Alignment of the intergenic region between YALI0F01628g and YALI0F01606g in *Yarrowia lipolytica* and strains from the *Yarrowia* clade highlighting conserved blocs that represents putative regulatory elements for the expression and regulation of the YALI0F01606g gene by erythritol and erythrulose.* Boxed* CAT and ATG correspond to the Stop and start codon of the YALI0F01628g and YALI0F01606g, respectively. *Cla*I and *Spe*I restriction sites are* underlined*. Localization of p300 primer containing the *Cla*I site is indicated above the genomic sequence. Genomic sequences are from *Y. lipolytica* W29 (YALI; YALI0F01606g), *Yarrowia phangngensis* (YAPH), *Yarrowia yakushimensis* (YAYA), *Yarrowia alimentaria* (YAAL), and *Yarrowia galli* (YAGA). Sequences are in Additional file 1: Table S1. The *Cla*I site upstream of the p300 sequence and the *Spe*I sites are* underlined*. The region containing UAS1_eyk_ used for tandem repeats construction is boxed

In order to identify the regulatory element (i.e. UAS) of pEYK1, we analysed the nucleotide sequence of the *EYK1* promoter region using the intergenic region between YALI0F01628g and YALI0F01606g (Fig. 5; Additional file 1: Table S1). Blast analysis of the *EYK1* promoter did not evidenced any conserved motif within *Yarrowia lipolytica* genome (data not shown). Therefore, we compared the promoter region of the *EYK1* gene to those present in other species of the *Yarrowia* clade (namely, *Yarrowia phangngensis*, *Yarrowia yakushimensis*, *Yarrowia alimentaria* and *Yarrowia galli* that have been recently sequenced and annotated in our laboratory [47] and Neuveglise N., Devillers H. et collaborator (unpublished). Alignment of the *EYK1* promoter sequences (Fig. 5; Additional file 1: Table S1) highlighted three putative conserved elements; a putative TATA box (Box TATA) and a conserved A motif (Box A) with the main signature [GGAAAGCCGCY] and a conserved B motif (Box B) with the main signature [CNTGCATWATCCGAYGAC].

**Fig. 6:**
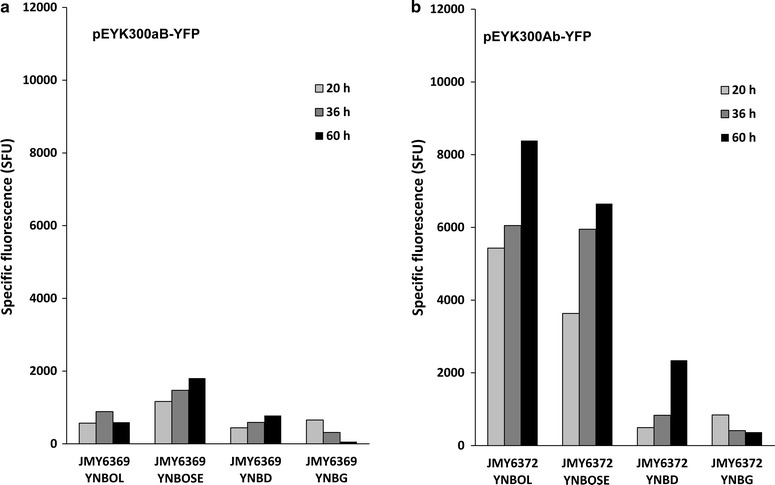
Time course of YFP fluorescence depending on culture medium for mutated pEYK300 promoter. Specific fluorescence (SFU) corresponding to the expression of YFP under: **a** promoter pEYK300 with a mutated A box; pEYK300aB (JMY6369) and **b** promoter EYK300 with a mutated B box; pEYK300Ba (JMY6372). Growth in minimum media YNB containing 1% of specified carbon source (*OL* erythritol, *EOSE*: erythrulose *D* dextrose, *G* glycerol)

The comparison of YFP fluorescence under pEYK450 and pEYK300 indicates that the TATA box may be involved in the expression of gene YALIPF01628g rather than gene YALI0F01606g. Thus, to determine the role of Box A and Box B in pEYK regulation, two mutated promoters, namely pEYK300aB and pEYL300Ab, were constructed as described in material and method by exchange of the *Cla*I-*Spe*I fragment. Mutation of the conserved Box A and Box B were performed by introducing a *Mul*I site. The motif A [GGAAAGCCGCC] was replaced by [GGA*ACGCGT*CC] and named motif a. The motif B [CTTGCATAATCCGATGAC] was replaced by [CTTG*T* *ACGCGT* *A*GATGAC] and named motif b. This yielded to pEYK300aB and pEYK300Ab, respectively (Fig. 2b). The mutated pEYK300aB and pEYK300Ab were introduced into strain JMY2101 (Po1d Leu^+^) to give rise to representative strains JMY6369 and JMY6372, respectively (Table 1). For strain JMY6369 carrying the pEYK300aB mutant promoter, YFP fluorescence was remarkably reduced in the presence of erythritol (YNBOL) and erythrulose (YNBOSE) (683 and 1481 mSFU, respectively) (Fig. 6a). This observation suggested that the Box A corresponds to the upstream activating sequence (UAS1_EYK1_) required for the promoter induction by both erythritol and erythrulose.

On the opposite, the mean relative YFP fluorescence measured for strain JMY6372 carrying the pEYK300Ab mutated promoter (Fig. 6b), was 2.4-fold higher in the presence of erythritol (YNBOL medium) than for the non-mutated pEYK300 promoter in the same conditions (8389 and 3536 SFU after 60 h, respectively). In contrast, YFP fluorescence in the presence of erythrulose (YNBOSE medium) was in the same range as YFP fluorescence of the non-mutated promoter. Furthermore, pEYK300Ab was less repressed on glucose media as compared to pEYK300 (with a mean specific fluorescence of 718 versus 279 mSFU), suggesting that the B box may be involved in glucose repression. This clearly demonstrates that domain A is involved in erythritol and erythrulose induction and that domain B may be involved in glucose repression since expression of the pEYK300Ab increased at the end of the culture in glucose media, which is not the case in glycerol media.

### Tamdem repeats of UAS1_EYK1_ increase promoter strength

Multicopy repeats of UAS elements upstream of a promoter have been shown to increase promoter strength [30, 31, 34, 48]. Therefore, we constructed promoter pEYK300A3B composed of three repeats of the 48 bp UAS1_EYK1_ fragment encompassing the Box A (GGGAAGCGGAATCCCAAAAG**GGAAAGCCGC**CGCATTAAGCTCCACAGC) upstream of the wild-type pEYK300 promoter (Fig. 2c). The resulting construct was introduced into strain JMY2101 to give rise to strain JMY6681.

**Fig. 7:**
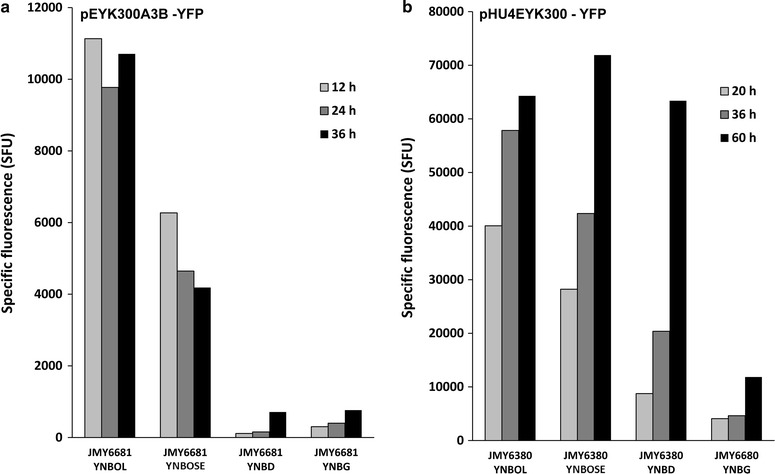
Time course of YFP expression depending on media for EYK hybrid promoters. Specific fluorescence (SFU) corresponding to the expression of YFP under: **a** pEYK300 A3B (JMY6681) and **b** pHU4EYK300 (JMY6380). Growth in minimum media YNB containing 1% of specified carbon source (*OL* erythritol, *OSE* erythrulose, *D* dextrose, *G* glycerol)

Promoter strength was monitored in the presence of glucose (YNBD), glycerol (YNBG), erythritol (YNBOL) and erythrulose (YNBOSE) and compared to that of pEYK300 (strain JMY6245). As shown in Fig. 7a, YFP fluorescence measured for pEYK300A3B was 3.4- fold higher in average in the presence of erythritol as compared to pEYK300 (10,538 and 3157 mSFU, respectively). In contrast, induction of pEYK300A3B was found similar in average in the presence of erythrulose as compared to pEYK300 (5034 and 4844 mSFU, respectively). By contrast to previous observation with pEYK300 (Fig. 4), pEYK300A3B induction level was 2.1-fold higher in average in the presence of erythritol than for erythrulose (10,538 and 5034 mSFU, respectively). Similar experiments performed with strain JMY6684 (pEYK300A3b), showed that the induction profile on YNBOL was not significantly different from the one of JMY6681 (pEYK300A3B), except that induction was significantly less repressed by glucose and glycerol, confirming the previous observations (data not shown).

Since the insertion of several copies of the 48 bp region encompassing the Box A motif resulted in a stronger promoter induction level, it could be assumed that increasing the copy number of UAS1_EYK1_ would allow to fine tune the strength of promoter induction. Indeed, several strong synthetic hybrid promoters have been created by fusing tandem repeats of upstream activation sequence (UAS) upstream to a core promoter region. The first one (hp4d) was based on four tandem repeats of the 108 bp UAS1_XPR2_ of the *XPR2* gene upstream on the minimal *LEU2* core promoter [30]. Later Blazek and coworker’s constructed hybrid promoters containing up to 32 copies of UAS1_XPR2_ of the *XPR2* gene upstream on the minimal *LEU2* core promoter and 16 copies of UAS1_XPR2_ of the *XPR2* gene upstream of TEF core promoters of different length [31]. Promoter strength increased with copy number of the UAS, and the best one showed a tenfold increase expression compared to the pTEF promoter. Similar expression levels were obtained by inserting three tandem copies of the 230 bp UAS1_TEF_ upstream of the pTEF promoter [48] and its expression did not vary significantly with carbon source (glucose, sucrose, glycerol and oleic acid). The only strong inducible promoter is the POX2 one [22]. Oleic acid inducible hybrid synthetic promoters were obtained comprising eight copies of UAS1_xpr2_ upstream of the 100 bp proximal core POX2 promoter. This UAS-core promoter chimera showed a 4.2-fold higher expression level in oleic acid media than in glucose in contrast to a twofold higher expression level for the 8 copies of UAS1_xpr2_ upstream of the 136 bp proximal core TEF promoter [34]. Here we showed that a hybrid promoter containing two additional tandem copies of the short 48 bp UAS1_EYK1_ upstream of the EYK1 promoter results in a 3.3-fold stronger promoter, thus we could expect to be able to construct stronger erythritol/erythrulose inducible promoters by introducing additional tandem repeats of the UAS1_EYK1_.

### UAS1B from XPR2 enhanced promoter strength without affecting erythritol and erythrulose induction

Madzak and colleagues reported that the fusion of four tandems repeats of UAS1B of *XPR2* gene upstream of a minimal promoter of the *LEU2* gene (yielding the so-called hp4d hybrid promoter) allowed a significant transcriptional activity [30]. In the same line, we combined four copies of UAS1_XPR2_ (UAS1B) with the pEYK300 promoter leading to promoter HU4EYK300 (JME3998) (Fig. 2c). The latter was introduced into JMY2101, giving rise to strain JMY6380. The regulation of the pHU4EYK300 was investigated by monitoring cell growth and YFP fluorescence levels during culture of strain JMY6380 in YNB medium supplemented with erythritol (YNBOL), erythrulose (YNBOSE), glucose (YNBD) and glycerol (YNBG). As shown in Fig. 7b, YFP fluorescence, and therefore promoter induction were 17.1- and 9.8-fold higher in the presence of erythritol (YNBOL medium) and erythrulose (YNBOSE) than for pEYK300 promoter (54,063 and 47,487 mSFU as compared to 3157 and 4844 mSFU, respectively). pHU4EYK was induced in stationary phase (i.e. after 60 h of culture, 63,380 SFU) on glucose media (YNBD) in contrast to pEYK300 (344 SFU). Nevertheless, pHU4EYK was not found highly expressed on glycerol media. Since pHU4EYK promoter yielded much stronger induction and thus gene expression, its regulation was further characterised in regard to the cell growth rate of strain JMY6380 and to the induction effect of erythritol and erythrulose on pHU4EYK.

### Hybrid promoter HU4EYK300 is inducible by erythritol and erythrulose

In order to further characterise the regulation of the hybrid promoter pHU4EYK300, its regulation was analysed at steady state in chemostat culture. This ensures that once the steady state is established, the effect of any perturbations—e.g., the addition of a known amount of a specific compound (an inducer or a repressor) in the medium—on pHU4EYK300 induction can be specifically assessed over time. The regulation of pHU4EYK300 was investigated in regard to the growth rate of strain JMY6380 and the composition of the culture medium, more specifically in the presence of a mixture of glycerol/erythritol or glycerol/erythrulose.

### *Growth rate has no effect on* pHU4EYK300 *induction*

Yeast cell physiology is directly influenced by the growth rate. With the aim to evaluate the influence of cell growth rate on pHU4EYK300 induction by erythritol, chemostat cultures were performed in YNBOL medium at two distinct dilution rates (i.e. 0.16 and 0.08 h^−1^). The fluorescence levels of YFP were monitored by flow cytometry to assess the induction level at the single cell level. No significant difference in the promoter induction levels could be observed for the two dilution rates tested (data not shown). Indeed, the mean relative fluorescence of the cell population was equal to 8.86 ± 0.62 × 10^4^ RFU at D = 0.16 h^−1^, and to 9.47 ± 0.31 × 10^4^ RFU at D = 0.08 h^−1^. Moreover, cytograms showed that the cell population is homogenously induced in presence of erythritol (Additional file 2: Figure S1).

#### Erythritol and erythrulose concentration modulate the strength pHU4EYK300 induction in the presence of glycerol

To assess the influence of inducer concentration on the regulation of pHU4EYK300, chemostat cultures of JMY6380 were performed on YNBG medium at a dilution rate of 0.2 h^−1^. At steady state, different amounts of erythritol or erythrulose were injected in the bioreactor to reach a final concentration of 0.2 and 0.6% (hereafter 0.2 CSP and 0.6 CSP), respectively. Glycerol, erythritol, erythrulose and YFP fluorescence were monitored for 8 h after inducer addition. In all experimental conditions tested, glycerol concentration remained almost constant (i.e. 3 g/L) in the bioreactor, confirming that a steady state was maintained in those experimental conditions.

**Fig. 8:**
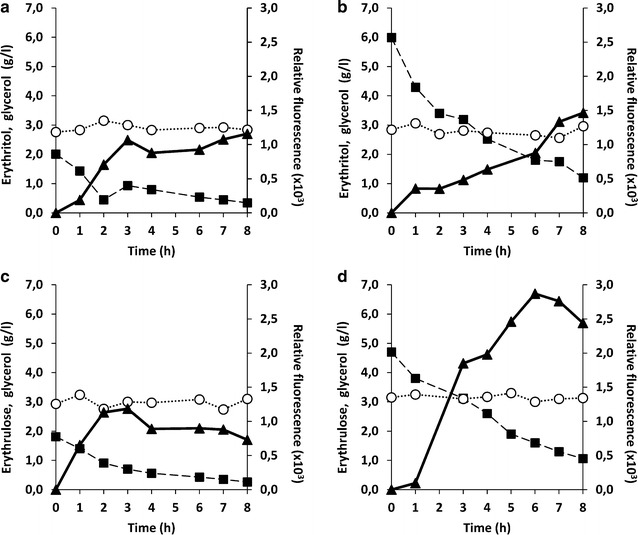
Induction of hybrid *EYK1* promoter pHU4EYK300 in continuous culture by erythritol (**a**,** b**) and by erythrulose (**c**,** d**). Erythritol or erythrulose and glycerol concentrations in the culture medium, and relative fluorescence of the cells during chemostat of JMY6380 (pHU4EYK300) on YNB-glycerol medium (1% glycerol). **a** Induction with a pulse of 0.2% of erythritol. **b** Induction with a pulse of 0.6% of erythritol. **c** Induction with a pulse of 0.2% of erythrulose. **d** Induction with a pulse of 0.6% of erythrulose. Time 0 corresponds to the time of the pulse.* Symbols* are: *black filled square*: erythritol or erythrulose; *open circle*: glycerol; *black filled triangle*: relative fluorescence (×10^3^). Figure illustrates representative experiments

As shown in Fig. 8, pHU4EYK300 induction level seems to be modulated by the inducer concentration in those experimental conditions (i.e. in the presence of glycerol). For 0.2 CSP, induction increased during the three first hours after inducer (erythritol and erythrulose) addition (Fig. 8a, c). After, when the inducer concentration was below 1 g/L, it remained almost constant for the next 6 h. By contrast, for 0.6 CSP, induction increased almost linearly during 8 h after inducer addition (Fig. 8b, d). It is worth mentioning that the amplitude of induction also seems to be correlated to the inducer concentration. The maximal YFP fluorescence and thus pHU4EYK300 induction, obtained after 8 h of erythritol addition was higher for the 0.6 CSP than for the 0.2 CSP (1.4 × 10^3^ and 1.1  × 10^3^ RFU, respectively). Similar observations were made for erythrulose. The maximal YFP fluorescence obtained 8 h after erythrulose addition was higher for the 0.6 CSP than for the 0.2 CSP (2.5 × 10^3^ and 1.1 × 10^3^ RFU, respectively). It could also be deduced from Fig. 8, that erythrulose yields to higher induction level than erythritol, even in the presence of 3 g/L of glycerol. These results obtained from a chemostat experiment confirm the observations made in Fig. 7b, i.e. pHU4EYK300 is a strong inducible promoter, responding to erythritol and even more to erythrulose as an inducer.

### Deletion of *EYK* enhanced pEYK expression

Erythritol and erythrulose could be used by *Y. lipolytica* as main carbon source (Fig. 1). Although glycerol was found to repress *EYK1* promoter, experiments in chemostat demonstrated that a mixture of glycerol/erythritol or glycerol/erythrulose could be used for induction. Moreover, both erythritol and erythrulose induced pHU4EYK300 promoter in a dose-dependent manner (Fig. 8). Therefore, we hypothesise that pEYK expression could be enhanced by deletion of the *EYK1* gene, thus erythritol and erythrulose could serve as inducers while avoiding their use as carbon sources for growth. Therefore, the auxotrophic *eyk1*∆ strain RIY176 (Table 2) was constructed as described in “Methods”. The expression cassette carrying pEYK300-YFP-*LEU2ex* was then introduced into RIY176, giving rise to strain RIY180 (JMY6637). Since *eyk1*∆ could not grow on erythritol and erythrulose as sole carbon source, strain RIY180 was grown in the presence of glucose or glycerol, used as energy source. Therefore, JMY6245 (pEYK300-WT) and RIY180 (pEYK300-*eyk1*∆) were grown in YNBDOL (glucose, erythritol), YNBGOL (glycerol, erythritol), YNBDOSE (glucose, erythrulose), or YNBGOSE (glycerol, erythrulose). Induction of the promoters was followed during time in microplates with glucose or glycerol for growth (0.25%) and with erythritol or erythrulose for induction (0.25%).

**Fig. 9:**
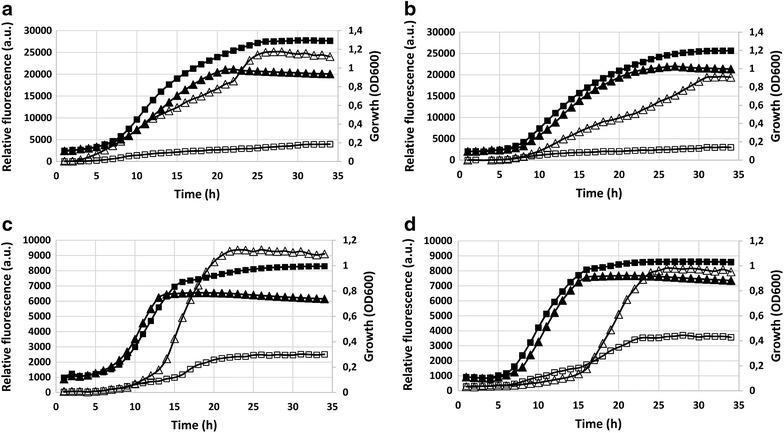
Time course of YFP fluorescence in wild-type and *eyk1*∆ strain under pEYK300. YFP fluorescence under pEYK300 in wild-type and *eyk1*∆ mutant, JMY6245 and JMY6638, respectively. Growth in minimum media YNB containing 0.25% of carbon source and 0.25% of inducer. **a** Glucose and erythritol. **b** Glycerol and erythritol. **c** Glucose and erythrulose. **d** Glycerol and erythrulose.* Symbols* are wild-type (*square*) and mutant (*triangle*). Growth (*full symbols*) and fluorescence (*empty symbols*)

As shown in Fig. 9, YFP expression in wild-type and *eyk1*∆ strains in the presence of erythritol occurred during the growth phase in media containing 0.25% of glucose or 0.25% of glycerol (Fig. 9a, b). YFP fluorescence at 34 h of growth was 8.3- and 7.8-fold higher in the *eyk1*∆ strain compared to the wild-type strain in glucose and glycerol, respectively (25,672 SFU versus 3078 SFU with glucose and 19,478 SFU versus 2500 SFU with glycerol). In contrast, in the presence of erythrulose, YFP expression in wild-type and *eyk1*∆ strains was somewhat delayed from the growth phase in media containing 0.25% of glucose or 0.25% of glycerol (Fig. 9c, d). However, YFP fluorescence was 4.9- and 2.6-fold higher in the *eyk1*∆ strain compared to the wild-type in glucose and glycerol, respectively (9106 SFU versus 2993 SFU with glucose and 7934 SFU versus 3564 SFU with glycerol).

For strain JMY6245 (pEYK300-WT), the rates of increase of YFP fluorescence in the presence of erythritol were 97 and 83 FU/h in glucose and glycerol, respectively. In comparison, in the mutant *eyk1*∆, the rates of increase of YFP fluorescence were 10.5-fold higher (1034 and 875 FU/h in glucose and glycerol, respectively).

Similarly, in the presence of erythrulose, higher induction levels were obtained for the *eyk1*∆ mutant (pEYK300-*eyk1*∆) as compared to the non-disrupted mutant (pEYK300-*EYK1*). The rate of YFP production in the mutant strain was 6.1-fold higher in glucose as compared to the wild-type strain (4000 and 347 FU/h, respectively). In the presence of glycerol, this increase was 7.3-fold (2527 and 875 FU/h, respectively).

These results demonstrate that expression levels could be further improved by using a strain deleted for the *EYK1* gene. In such a strain, erythritol or erythrulose could be used as a free inducer and independently from growth, for having induction either during the growth phase or delayed from this latter.

## Conclusions

Several groups have constructed hybrid promoters based on combination of repeats of upstream activating sequence (UAS), TATA box and core promoter for gene expression in *Y. lipolytica* [30, 31, 34, 48]. This gave rise to hybrid promoters with various strengths, up to tenfold higher expression than the constitutive pTEF promoter [32], this later one being a constitutive strong promoter commonly used for gene expression and for promoter strength comparison. Among them are few strong inducible promoters such as pICL1, pLIP2, pPOX2 [23–25, 49]. The LIP2 and POX2 promoters are inducible by oleic acid which has the drawback to require oil emulsion for induction. This study has identified a new short promoter (136 bp) inducible by erythritol or by erythrulose. Furthermore, promoter comparison allowed to identify a very short (43 bp) upstream activating sequence (UAS1_EYK1_) and a potential upstream regulatory sequence (URS_EYK_). This study has generated new hybrid promoters combining core EYK promoter with either UAS1_EYK1_ or UAS1_XPR2_ upstream activating sequences, allowing at least a tenfold higher expression than the pTEF promoter. This opens the path to the design of new synthetic promoters containing UAS_EYK_ and/or URS_EYK_ with higher tandem repeats number or with various core promoters to further widen the expression range and the induction profiles.

These promoters are poorly expressed in glucose or glycerol and could be induced by erythritol or by erythrulose with a tremendous advantage of being dose dependant thus allowing fine tuning of induction which will permit to vary the degrees of expression that could be obtained. One would be easily able to regulate the expression level depending on UAS1_EYK1_ copy number, the induction time depending on the inducer used (erythritol or by erythrulose), the induction level depending on the media and the inducer concentration. One will have also to choice using erythritol or erythrulose as inducer and source of carbon for growth or use only as inducer in a ∆*eyk1* genetic background.

These inducible promoters and UAS1_EYK1_ expand the parts available for protein expression [33] and for the development of tools for genetic engineering such as additional marker for gene deletion or marker rescue [40, 41] and for inducible expression of gene such as CAS9 for genome editing [50, 51]. These new promoters could be also a powerful tool for fundamental research as was the development of the GAL1 promoter in *Saccharomyces cerevisiae* [52].

## Additional files

**Additional file 1.** Sequence of the upstream region of the *EYK1* genes in *Yarrowia* clade. Sequence in *Y. lipolytica* (YALI-pEYK1), *Yarrowia phangngensis* (YAPH-pEYK1), *Yarrowia yakushimensis* (YAYA-pEYK1), *Yarrowia alimentaria* (YAAL-pEYK1), and *Yarrowia galli* (YAGA-pEYK1). Underlined are the nucleotidic sequences of the end of YALI0F01628g with the stop codon (CAT) and at the beginning of YALI0F01606g with the start codon (ATG).

**Additional file 2.** Influence of dilution rate on the induction of hybrid EYK1 promoter pHU4EYK300 in continuous culture by erythritol. FL1-A/FSC-A cytograms corresponding to the chemostat of JMY6380 (pHU4EYK300) on YNB-erythritol medium (1% erythritol). The horizontal line at 4x10^3^ FU represents the limit between induced cells (quadrant Q1-UR of the cytogram) and non-induced cells (quadrant Q1-LR of the cytogram). Cytograms are representative of two independent cultures, and are the result of the analysis of 40,000 cells. **a** Cytogram of the equilibrium cell population cultivated at a dilution rate of D = 0.16 h^−1^. **b** Cytogram of the equilibrium cell population cultivated at a dilution rate of D = 0.08^−1^.

## Abbreviations

CSP: carbon source pulse
D: dilution rate
EYK1: erythrulose kinase
FU: fluorescence units (flow cytometry)
GRAS: generally recognized as safe
Leu/LEU2: 3-isopropylmalate dehydrogenase gene
PCR: polymerase chain reaction
SFU: specific fluorescence units (Biolector)
mSFU: mean specific fluorescence
UAS: upstream activating sequence
Ura/URA3: orotidine-5′-phosphate decarboxylase
Vvm: volume (of gas) per volume (of culture medium) per minute
YFP: yellow fluorescent protein
YNBD: YNB + glucose
YNBG: YNB + glycerol
YNBOL: YNB + erythritol
YNBOSE: YNB + erythrulose
YNBDOL: YNB + glucose + erythritol
YNBGOL: YNB + glycerol + erythritol
YNBDOSE: YNB + glucose + erythrulose
YNBGOSE: YNB + glycerol + erythrulose
