# Supplementary material for: New inducible promoter for gene expression and synthetic biology in Yarrowia lipolytica
Source: Microb Cell Fact. 2017 Aug 15;16:141. doi: 10.1186/s12934-017-0755-0 (PMC5557077; doi:10.1186/s12934-017-0755-0)
Supplement: Supplementary file 1 — Additional file 1. Sequence of the upstream region of the EYK1 genes in Yarrowia clade. Sequence in Y. lipolytica (YALI-pEYK1), Yarrowia phangngensis (YAPH-pEYK1), Yarrowia yakushimensis (YAYA-pEYK1), Yarrowia alimentaria (YAAL-pEYK1), and Yarrowia galli (YAGA-pEYK1). Underlined are the nucleotidic sequences of the end of YALI0F01628g with the stop codon (CAT) and at the beginning of YALI0F01606g with the start codon (ATG). [file 12934_2017_755_MOESM1_ESM.docx]

**Table S1**. Sequence of the upstream region of the *EYK1* genes in *Yarrowia* clade. Sequence in *Y. lipolytica* (YALI-pEYK1), *Yarrowia phangngensis* (YAPH-pEYK1), *Yarrowia yakushimensis* (YAYA-pEYK1), *Yarrowia* *alimentaria* (YAAL-pEYK1), and *Yarrowia* *galli* (YAGA-pEYK1). Underlined are the nucleotidic sequences of the end of YALI0F01628g with the stop codon (CAT) and at the beginning of YALI0F01606g with the start codon (ATG).

| YALI-pEYK1 | TCCAACAACAATAGTGAATCC**CAT**TTTGTGCAAGTGTGTGTGTGTGTGTGTGTGTGGTGTGTTTGTGTGTTAGAACGGATGTTCTGGTGAGTGTGAGTGTGTAGTTGTGTGATGAGACCTTGGTGCCACCCCAAGGTATATATATATAACACCTCCAGGAGCTCTAAAAAGGCATCTACTTTTCTCTATACTGTACGTTTCAATCTGGGGAAGCGGAATCCCAAAAGGGAAAGCCGCCGCATTAAGCTCCACAGCCTTGCATAATCCGATGACCTGACTAGTGCGGACAAAGACTATTATTTCGAGGCAAGGCCACCACGTACCGCGGTCCCAAACTTTTGCAAAGCTGAAAACAGCGTGGGGGTCAACGTGGATCAGAAAGAGGGGCAGATCAGCTTCTATAAGAAGCTCCTTTCCCCACAATTGGCCCACACGACACTTCTACACACTTACACATCTACT**ATG**TCCACAAAACATCTGTTCAACGA |
| --- | --- |
| YAGA-pEYK1 | TCCAACAACAATAGTGAATCC**CAT**TTGTGTGTGTGTGTGTGTGTAAGAGTGGTTTTGTGTGTGTTAGAACGGTTGTTCTGTTGTGTGTGTTTGTGTAGTTTGTGTGTTTGTGTCGTTCTGGCCTGGCCAAGGTATATATACCACCAACTGTCTTACCAAGAGCTCTAAAGACCTCCTCAGCCATTCAGTATAGTACGTTTCAATCCGGGGAAGCGGCATCCCAATTGGGAAAGCCGCCGCATTAAGCTCCACAGCCTTGCATAATCCGATGACCTGACTAGTGCAGACAAAGACTTTAATTTTTCGAGGCAAGCACACTACACACAACAACCACGTATCGTGCCAAACTTTTGCAAAACAGAAAACGCCAGAGTGGGGGTCAAATTGGCTTGGATCAGGAAGGGAGAAAGAGGGGCTGTCGGGATCAGCTTCTATAAGAACCTGTCTTCCCCCAATTGGTCCAGATCAACCAACTACACACAACTACACCACAACTACAA**ATG**TCCACCAAACACCTGTTCAACGA |
| YAYA-pEYK1 | TCCAACAACAATGATGAATCC**CAT**AGTAGTGTAAGGTGTTGGCGTGTGTGTTTGAAGTGACCAGACCACACAAAGGTATATATATCACCGCTACGAGCTCCTCAAACAGTCCTTCTTTCAGTCTACGGAATCCTGAAATGGAAAGCCGTCACATAAACTTTTTAATCATGCATAATCCGATGACCTGACTGGTCCGACGGAGTCTATTTTTTTTTCAGACAATGACTTTTCGATACTAACCCACTCGTAGCGTGGATCCGTAAACAGAGAGTGGGGGTCAGGGATCGGAAAGAGGGGCACATTGTTCCTATAAGACCGTGTATCCCCAAAATCTCAACCTCAACATTCACCCAAAGAC**ATG**TCTACAAAACACCTATTCAACGA |
| YAAL-pEYK1 | ACCAACAACAATTGTGAATCC**CAT**TTTGAATAATGTCTTGTGTGTAGTGAGTGGTTTGTGAGGGAGGTTGTGTTCTGTTGTACGTTGTGTGCTCTATACGGGCGGCCTAGTATATATATATAACTCCTAAGGCAGACCCAAAGGCACCCCCACAGTCTTTTCCCACGTCCTGTCAGTAACAAGGCCCGGGCATGCTCTCATTTTCTGGGGAAGGGTGCGGAATCCCAAAAGGGAAAGCCGTCCCAATAAACTCAATAAACGTGCATAATCCGATGACCTGACAAGCAGACAAAGACTAATTTTCCAAGCAAGTGCCGAAAGTGCCAATACCCACGCTATGTGTACGTGGGGGCACCAGAAGACCCAAAGAGGACCCGACAGATCTATAAAGACTCTCTCGCCACTCACCCTTCTCATCCACCAACACACAAACA**ATG**TCCACCAAACATCTTTTCAACGA |
| YAPH-pEYK1 | TCCAACAACAATTGTGAATCC**CAT**TTTATTTTGTAGTGTCTGATAAAAGTTGAGTGATTGGTTTCCAGTGGGGAAGAGGTTATTCTTGTAGTCGGAGCTTTTTGATCCCAGCACTATTTATAACACAATTACAATCTGTCTGTCTCTAAATTCATACTTTCCGTAAATTCCTATTACGATTTATTCTAGTCCCTTTCGCGGAACCTCAAAGTGGAAAGCCGTGGAATTAAGCTCATAAAGCTTGCATTATCCGACGACAATACAAACCTTAACTCGAACAAAGGAATTCATGGGGACATTTACGTGGGGGCCGGTCTAACTGTGCTATTGAGTGTCCCGTCAGAAAAAATGCTATAAAGGATGATTCTCCTCTCTTTCCTTTTTGTCTCTCATCAATTCTGATTACGGTTCACAAC**ATG**TCAACAAAGCATCTCTTTAACGA |
