# Supplementary figures and images for: New inducible promoter for gene expression and synthetic biology in Yarrowia lipolytica
Source: Microb Cell Fact. 2017 Aug 15;16:141. doi: 10.1186/s12934-017-0755-0 (PMC5557077; doi:10.1186/s12934-017-0755-0)

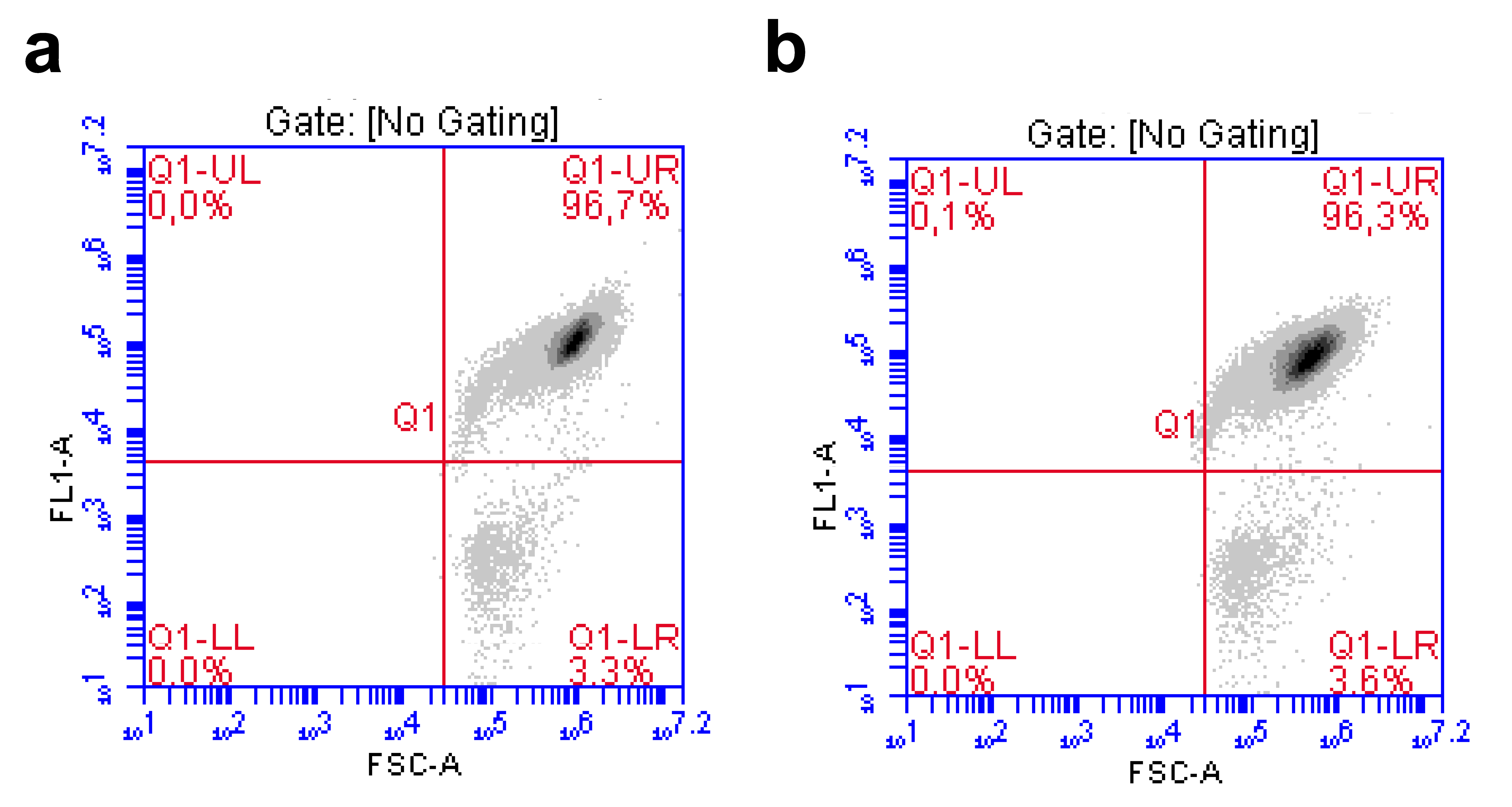

Supplement: Supplementary file 2 — Additional file 2. Influence of dilution rate on the induction of hybrid EYK1 promoter pHU4EYK300 in continuous culture by erythritol. FL1-A/FSC-A cytograms corresponding to the chemostat of JMY6380 (pHU4EYK300) on YNB-erythritol medium (1% erythritol). The horizontal line at 4x10^3^ FU represents the limit between induced cells (quadrant Q1-UR of the cytogram) and non-induced cells (quadrant Q1-LR of the cytogram). Cytograms are representative of two independent cultures, and are the result of the analysis of 40,000 cells. a Cytogram of the equilibrium cell population cultivated at a dilution rate of D = 0.16 h−1. b Cytogram of the equilibrium cell population cultivated at a dilution rate of D = 0.08−1. [file 12934_2017_755_MOESM2_ESM.tif]
